# Vaccine Protection Through Placenta and Breastfeeding: The Unmet Topic in COVID-19 Pandemic

**DOI:** 10.3389/fimmu.2022.910138

**Published:** 2022-06-03

**Authors:** Ariane Laguila Altoé, Anna Paula Marques Mambriz, Daniela Maira Cardozo, Joana Maira Valentini Zacarias, Jeane Eliete Laguila Visentainer, Larissa Danielle Bahls-Pinto

**Affiliations:** ^1^ Department of Basic Health Science, Laboratory of Immunogenetics, State University of Maringa, Maringa, Brazil; ^2^ Department of Medicine, State University of Maringa, Maringa, Brazil; ^3^ Department of Clinical Analysis and Biomedicine, Postgraduate Program in Biosciences and Physiopathology, State University of Maringa, Maringa, Brazil; ^4^ Clinical Hospital, State University of Campinas, Campinas, Campinas, Brazil

**Keywords:** COVID-19, SARS-CoV-2, vaccine, human milk, passive immunization

## Abstract

The coronavirus disease 2019 (COVID-19) pandemic has turned pregnant women’s healthcare into a worldwide public health challenge. Although initial data did not demonstrate pregnancy as a more susceptible period to severe outcomes of acute severe acute respiratory syndrome-related coronavirus 2 (SARS-CoV-2) infection, there are an increasing number of reports showing that not only pregnant women might be at significantly higher risk than non-pregnant women by COVID-19 but also the fetus. These findings may be related to adaptive changes that occur during pregnancy, such as the reduction in the residual respiratory capacity, the decrease in viral immune responses, and the increased risk for thromboembolic events. Additionally, despite the SARS-CoV-2 vertical transmission evidence being uncommon, maternal illness severity might reflect serious perinatal and neonatal outcomes. Thus, protecting the maternal–fetal dyad against COVID-19 is critical. Even though pregnant women initially were excluded from vaccine trials, several studies have provided safety and efficacy of the overall vaccine COVID-19 platforms. Vaccination during pregnancy becomes a priority and can generate benefits for both the mother and newborn: maternal neutralizing antibodies are transmitted through the placenta and breastfeeding. Moreover, regarding passive immunization, human milk contains other bioactive molecules and cells able to modulate the newborn’s immune response, which can be amplified after the vaccine. Nonetheless, many issues remain to be elucidated, considering the magnitude of the protective immunity transferred, the duration of the induced immunity, and the optimal interval for pregnant immunization. In this review, we assessed these unmet topics supported by literature evidence regarding the vaccine’s immunogenicity, pregnancy immune heterogeneity, and the unique human milk antiviral features.

## Introduction

In December 2019, a virus called severe acute respiratory syndrome-related coronavirus 2 (SARS-CoV-2) was identified in China (1). This new respiratory disease was named coronavirus disease 2019 (COVID-19) by the WHO, and in March 2020, it was declared a pandemic (2). The case fatality rate (CFR) of COVID-19 was estimated at 2.3% (3, 4), which is reflected in more than 475 million cases and 6.1 million deaths registered worldwide to date (5). This remarkable ability to spread is explained by the high viral transmissibility added to characteristics such as long incubation period, infectivity capacity before the beginning of symptoms, and a large number of asymptomatic cases and/or mild diseases (6). In fact, it is estimated that approximately 55%–60% of infected individuals present some symptom, and the majority of them (81%) develop mild disease (fever, cough, fatigue, dyspnea, myalgia, headache, and diarrhea); of the other infected individuals, 14% evolve to severe disease, and 5% develop the critical disease, frequently needing to stay in an intensive care unit (ICU) (3, 5). However, these statistics may be different in some risk groups such as frontline healthcare professionals; elderly people; patients with heart, pulmonary, or neurologic diseases; patients with diabetes mellitus, obesity, or immunosuppression; and pregnant/postpartum women (6).

Concerning pregnancy and lactation, although the initial studies involving pregnant women were not conclusive (7), a series of severe complications in pregnant women and their newborns have been associated with SARS-CoV-2 infection (8). This outbreak was expected as previous coronavirus pandemic diseases such as SARS and Middle East respiratory syndrome (MERS) had already presented similar risks for mother and child (5, 7, 9, 10). Thus, although early studies have shown that pregnant women have milder symptoms than non-pregnant women in SARS-CoV-2 infection (5, 11–13) and a lower incidence of gestational and neonatal complications (5, 14–16), growing evidence suggests that pregnant women diagnosed with COVID-19 are at increased risk for ICU admission and need for invasive ventilation/extracorporeal membrane oxygenation (ECMO), higher morbidity and mortality, and higher odds of maternal–fetal complications (such as preterm birth and miscarriage), thrombosis, intrauterine fetal growth, intrauterine transmission, congenital anomalies, and neurologic abnormalities) when compared to those without COVID-19 (8, 14–20).

**Figure 1:**
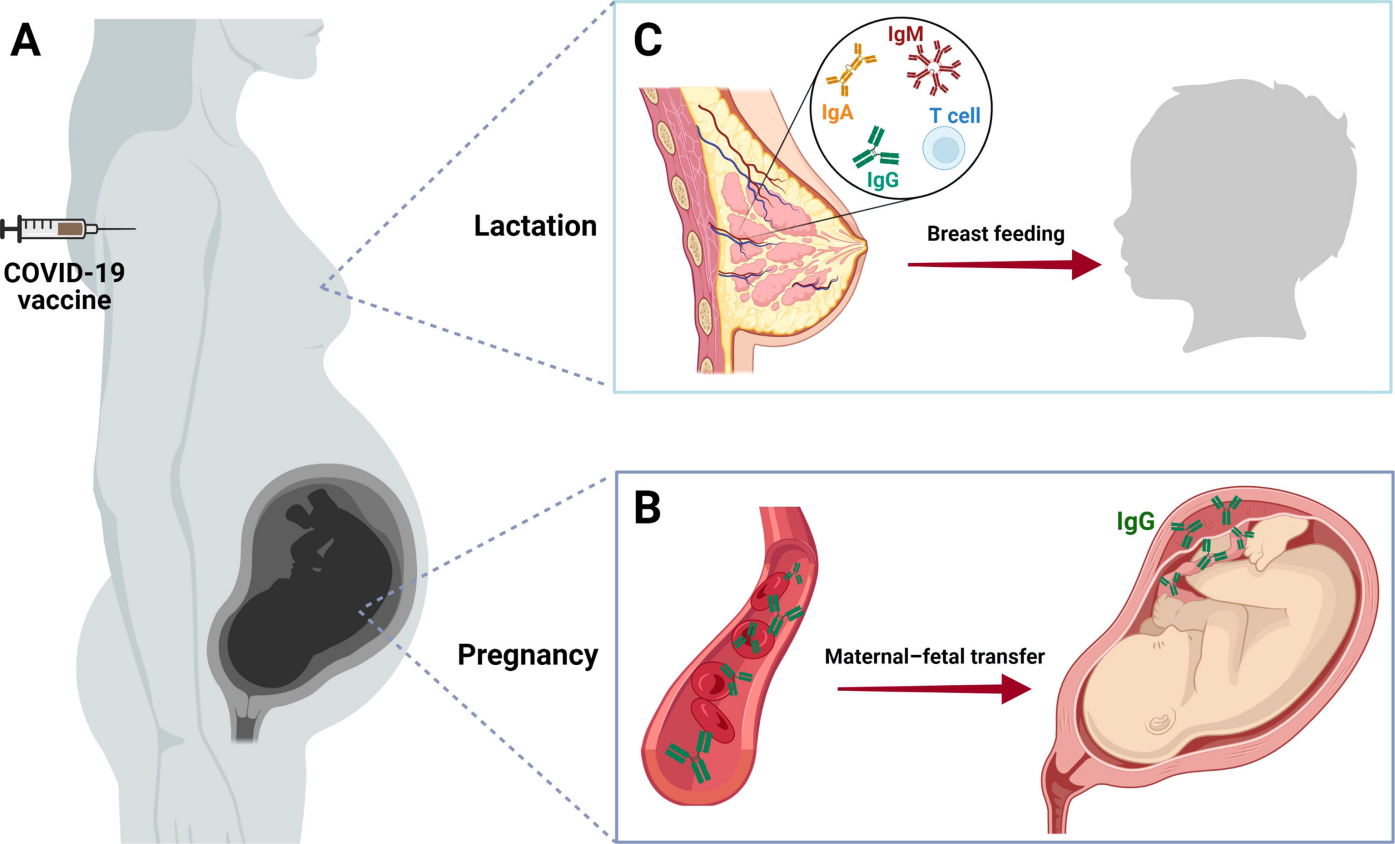
COVID-19 vaccine in pregnancy and lactation. **(A)** Two pathways of maternal–fetal protection against SARS-CoV-2 after COVID-19 vaccination. **(B)** After receiving the COVID-19 vaccine, pregnant women start to develop antibodies against the virus (IgG). Thus, immunized women are able to transmit anti-SARS-CoV-2 IgG molecules from their blood to the fetus. This process occurs passively through the placenta, and it is confirmed by the presence of these antibodies in cord blood or the newborn serum after birth. **(C)** Passive immunization of the newborn also happens through breastfeeding, which can be demonstrated by the presence of anti-SARS-CoV-specific IgA, IgM, IgG, and T cells in breast milk. These findings reinforce the importance of pregnant and lactating women to complete the vaccination schedule, protecting themselves and their infants from the severe manifestations of COVID-19. Created with BioRender.com.

In this mini-review, we summarize the last information about COVID-19 vaccines in use by pregnant women, with an emphasis on its immunogenicity in this particular group and on the transmission of the acquired immunity to the fetus and the newborns (**Figure 1**).

## Peculiarity of Immune System During Pregnancy

Pregnant women are usually considered at high risk for infectious diseases; it happens due to physiologic, cardiopulmonary, and immunologic changes in their bodies during pregnancy (3, 21). In this period, the diaphragm is pushed to a higher position as the uterus expands; this can create an obstacle for the lungs to expand, additionally the upper respiratory tract swells, and the oxygen demand increases. Thus, the intolerance to hypoxemia makes pregnant women more likely to develop respiratory disease complications, including COVID-19 (22, 23). In this period, marked by significant hormonal changes, a shift in the balance between T helper 1 (Th1)-mediated and T helper 2 (Th2)-mediated immunity can be observed: a decrease in Th1 response leads to a dominant Th2 humoral immune response, which results in a lower secretion of proinflammatory cytokines, such as interleukin-2 (IL-2), interferon-gamma (IFN-*γ*), and tumor necrosis factor-alpha (TNF-α) and an increase in anti-inflammatory cytokines (IL-4, IL-10, and IL-13), respectively (24, 25). Indeed, a decrease in NK cells and plasmacytoid dendritic cells (which compromises the production of type 1 IFN) and a decrease in phagocytic activity were observed. This scenario creates an immune tolerance for the fetus but increases the susceptibility of pregnant women to SARS-CoV-2 infection (26).

Despite this period of women’s life being marked mainly by an immunotolerant profile, actually pregnancy involves a triphasic immune modulation, characterized by an alternation between proinflammatory, anti-inflammatory, and a second proinflammatory state, in that order, over the three trimesters (22). Thus, women in the first and third trimester of pregnancy have a proinflammatory profile, and for this reason, when infected with SARS-CoV-2, they are more likely to develop the cytokine storm, leading to bad maternal and fetal prognoses (21, 23).

## Pregnant COVID-19 Vaccination: State of the Art

Fortunately, different kinds of COVID-19 vaccines are now available to the global population, and the evolution of epidemiological data has shown that they are essential for the control of SARS-CoV-2 spread and, especially, for the decrease of COVID-19 morbidity–mortality worldwide (27, 28). All the COVID-19 vaccines approved for use in the population are allowed during pregnancy if the benefits outweigh the possible risks (15, 16, 29, 30). Initially, two anti-COVID-19 vaccines, which use mRNA technology, were authorized: Pfizer/BioNTech (Germany and USA) and Moderna (USA) (31). The first one is administered in 2 doses, with 3 weeks of interval between them, and the second one also involves 2 doses, but with 4 weeks of interval. Both vaccines have about the same effectiveness, approximately 94.1% to 95% (32). Other than that, mRNA technology was also approved for three viral vector vaccines: Oxford-AstraZeneca (UK and Sweden), Sputnik (Russia), and Janssen (Belgium) (13, 32). The recommended administration is 2 doses for AstraZeneca, with an interval of <6 or >12 weeks between first and second doses (effectiveness from 55.1% to 81.3%); Sputnik uses 2 doses administered 3 weeks apart (effectiveness of 91.6%); Janssen was proposed as a single-dose vaccine (effectiveness of 66% against moderate to severe to critical COVID-19 and 76.7% to 85.4% against critical disease) (32). A sixth approved vaccine called Sinovac-CoronaVac (China, and lately produced by Instituto Butantan in Brazil) uses inactivated SARS-CoV-2 virus antigen and is administered in 2 doses (2–4 weeks apart between then; effectiveness of 83.7% against moderate disease to 100% against severe disease) (32, 33).

None of the approved COVID-19 vaccines contain a replicant virus; thus, they cannot cause the disease. Studies with animals did not demonstrate dangerous effects related to Pfizer, Moderna, AstraZeneca, Sputnik, and Janssen vaccines in pregnancy (13). In general, the side effects of vaccination are similar in pregnant and other groups, with non-specific side effects due to activation of the immune system being the most worrying (34). Although rare, some immune-mediated complications were already described, such as myocarditis/pericarditis after immunization with mRNA vaccines and Guillain–Barré syndrome and thrombotic events after viral vector vaccines (35–37). It is important to note the rare cases of post-COVID-19 vaccine thrombosis with thrombocytopenia syndrome (TTS) occur by a mechanism distinct from thromboembolic events that usually happen during pregnancy and post-childbirth (38–40). Moreover, according to a systematic review and meta-analysis recently published, there are no class-wide effects of adenovirus-based vaccines on thrombocytopenia or coagulopathy in pregnancy or the general population (41). Thus, after several investigations, authorities determined that adenovirus vector vaccines could be used by pregnant women, and the TTS occurrence probability is similar to that in the general population (42). The decision about the better choice between the abovementioned vaccine platforms should be discussed between the health professional and the pregnant/lactating woman, considering the effectiveness, security, and other parameters (43, 44). Still, there are few published data on the COVID-19 vaccine in pregnant women, mainly because they are not usually included in vaccine clinical trials due to safety and responsibility concerns (45); nevertheless, several studies support its safety and effectiveness (46, 47).

## Humoral Immune Response Post-COVID-19 Vaccine in Pregnant Women

Prospective cohorts revealed that anti-SARS-CoV-2 humoral and cellular responses are similar between immunized pregnant and non-pregnant women and more robust when compared to infected and unvaccinated individuals (43, 48–50); this proves that vaccination gives higher immunity than natural infection by SARS-CoV-2 (51). A study performed by Collier et al. showed that after receiving COVID-19 mRNA vaccine, both pregnant and non-pregnant women had their titers of IgG and IgA against the receptor-binding domain (RBD) from spike protein of SARS-CoV-2, and the titers of the pseudovirus neutralizing antibody (NT50) similarly increased (52). Another study that compared anti-SARS-CoV-2 IgG levels between mRNA COVID-19 vaccinated pregnant women and SARS-CoV-2 diagnosed pregnant women found that while vaccination increased levels of anti-S1 and anti-RBD IgG antibodies, the infection was associated with higher levels of anti-S2 and IgG neutralizing antibodies (46).

Additionally, Golan et al. demonstrated that serum levels of anti-SARS-CoV-2 IgM and IgG antibodies were significantly higher after the first dose of the mRNA vaccine; furthermore, the second dose significantly increased anti-SARS-CoV-2 IgG serum levels, but not anti-SARS-CoV IgM serum levels, characterizing a secondary immune response against the virus (53). These findings are in agreement with those found by Leik et al., who showed that the anti-spike IgG and anti-RBD IgG titers increased after the first dose of the vaccine but were much higher when pregnant women received the second dose (46). These results highlight the importance of the second dose to the development of higher titers of protective antibodies in pregnant women.

Another important topic related to vaccination is the durability of conferred protection. In this regard, studies demonstrated that approximately 5 to 6 months after taking the second dose of the SARS-CoV-2 vaccine, its effectiveness naturally starts to decrease (54–57). Thus, in order to recover the immune response against the virus, a booster dose has been recommended for some high-risk groups, including pregnant women (58). A recent study demonstrated that women who received the third dose in the last trimester of pregnancy presented higher levels of anti-spike IgG in maternal and cord blood (59), which suggests that women with a complete vaccination schedule (two initial doses followed by a booster dose) transmit a higher concentration of antibodies to the infant than those who only received the first and second doses. These findings indicate the benefits of early COVID-19 immunization protocol in pregnant women, which are sustained by results that demonstrate that COVID-19 vaccination during early pregnancy is not associated with an increased risk of fetal structural anomalies (60).

### Maternal–Fetal Anti-SARS-CoV-2 Antibody Transmission

The transmission of humoral immunity from mother to fetus or newborn throughout the placenta or human milk is well established. As explored below and summarized in **Table 1**, studies that investigated if it also occurs for anti-SARS-CoV-2 antibodies found that pregnant women who had COVID-19 active infection or received the COVID-19 vaccine developed anti-SARS-CoV-2 IgM, IgG, and IgA, and these antibodies were transferred to the fetus *via* placental transport or breastfeeding (48, 51, 53, 70–73).

**Table 1:** Anti-SARS-CoV-2 antibodies production and maternal–fetal transfer after COVID-19 vaccination.

References	Producer	Type of vaccine	N pregnant	N lactating	Antibodies researched	Main findings in serum	Main findings in breast milk	Main findings in umbilical cord
(48)	Pfizer-BioNTech	BNT162b2 mRNA	41	16	IgM, IgA, and IgG anti-spike, RBD, S1, and S2	1. Increase in all antibodies at the first and second doses 2. Significant increase in IgG at the third dose 3. Dominant IgG antibody response	1. Increase in all antibodies in the first and second doses and significant increase in IgG in the third dose 2. Increased transfer of IgG1 RBD at the third dose 3. IgG transfer *via* breast milk	1. Anti-spike IgG and RBD found in the cord 2. Transfer of IgG *via* the placenta
	Moderna-NIH	mRNA-1273	43	15	IgM, IgA, and IgG anti-spike, RBD, S1, and S2	1. Increase in all antibodies at the first and second doses 2. Significant increase in IgG at the third dose 3. Dominant IgG antibody response 4. More robust anti-spike and anti-RBD IgA response	1. Increase in all antibodies in the first and second doses and significant increase in IgG in the third dose 2. Increased transfer of IgG1 RBD at the third dose 3. IgG transfer *via* breast milk	1. Anti-spike IgG and RBD found in the cord 2. Transfer of IgG *via* the placenta
(61)	CoronaVac^®^	Inactivated Virus Antigen	1	0	Total Neutralizing Antibodies to SARS-CoV-2	Positive reaction for neutralizing antibodies in NB serum, 24 h after birth	NA	NA
(62)	Pfizer-BioNTech	BNT162b2 mRNA	0	14	IgM, IgA, and IgG anti-spike	Maternal IgG and IgM increased after second dose	IgG and IgA present in approximately 40% of samples	NA
(53)	Pfizer-BioNTech	BNT162b2 mRNA	0	19	IgA and IgG anti-RBD	1. High maternal IgG levels after second dose 2. IgG detection in babies whose mother was vaccinated during pregnancy but not after delivery	1. Higher levels of IgA 2. Increase in IgG after second dose	NA
	Moderna-NIH	mRNA-1273	0	13	IgA and IgG anti-RBD	1. High maternal IgG levels after second dose 2. IgG detection in babies whose mother was vaccinated during pregnancy but not after delivery	1. Higher levels of IgA 2. Increase in IgG after second dose	NA
(63)	Pfizer-BioNTech	BNT162b2 mRNA	0	70	IgA and IgG anti-RBD	Detection of IgG and IgA in the mother’s serum	Detection of IgG and IgA	NA
	Moderna-NIH	mRNA-1273	0	20	IgA and IgG anti-RBD	Detection of IgG and IgA in the mother’s serum	Detection of IgG and IgA	NA
	AstraZeneca	Replication-deficient simian adenovirus vector ChAdOx1-S	0	20	IgA and IgG anti-RBD	Lower detection of IgG and IgA in maternal serum	Lower detection of IgG and IgA	NA
(64)	Pfizer-BioNTech	BNT162b2 mRNA	0	21	Anti-spike SIgA, IgA, IgG, and IgM; spike T cells	Detection of IgA, IgG, and IgM	1. Detection of IgA, IgG, and IgM 2. Immune transfer to breast milk occurs through spike SIgA, IgG, and T cells	NA
	Moderna-NIH	mRNA-1273	0	2	Anti-RBD IgG, IgA, and IgM; spike T cells	Detection of IgA, IgG, and IgM.	1. Detection of IgA, IgG, and IgM 2. Immune transfer to breast milk occurs through spike SIgA, IgG, and T cells	NA
(65)	Pfizer-BioNTech	BNT162b2 mRNA	0	84	Anti-spike IgA and IgG	NA	Detection of IgA and IgG	NA
(66)	Pfizer-BioNTech	BNT162b2 mRNA	0	33	Anti-spike IgG	Detection of IgG	Detection of IgG	NA
(67)	CoronaVac^®^	Inactivated Virus Antigen	0	20	Anti-spike IgA	NA	Detection of IgA	NA
(68)	Pfizer-BioNTech	BNT162b2 mRNA	0	14	Anti-spike IgA and IgG	Detection of IgA and IgG	Detection of IgA and IgG	
	Moderna-NIH	mRNA-1273	0	7	Anti-spike IgA and IgG	Detection of IgA and IgG	Detection of IgA and IgG	NA
(69)	Pfizer-BioNTech	BNT162b2 mRNA	Not cited	25	Anti-spike IgM, IgG and IgA	Detection of IgA and IgG	1. Detection of IgA, IgG, and IgM 2. The SARS-CoV-2 antibodies induced by mRNA vaccines persist for at least 6 months	NA
	Moderna-NIH	mRNA-1273	Not cited	2	Anti-spike IgM, IgG and IgA	Detection of IgA and IgG	1. Detection of IgA, IgG, and IgM 2. The SARS-CoV-2 antibodies induced by mRNA vaccines persist for at least 6 months	NA

N, number of individuals; NA, not analyzed; NB, newborn.

Regarding the transplacental route of infant’s passive immunization, Leik et al. reviewed studies that demonstrated anti-spike IgG, anti-RBD IgG, and neutralizing IgG in blood samples of newborns of vaccinated women; furthermore, these antibody levels were higher among those whose mothers had received two doses of vaccine (46). Additionally, a recent paper showed that after a third dose, the levels of neutralizing antibodies against SARS-CoV-2 were higher in both mother blood and cord blood, strengthening the importance of a boost dose to increase humoral immune transfer to the newborn (59). Importantly, it was demonstrated that the majority of maternal IgG is transferred to the fetus in the last 4 weeks of gestation (70, 74). This information is crucial to better determine the administration period for this specific public, to ensure the protection of the newborns from possible infections. Thus, the seroprotection during the beginning of the infant’s life can be enhanced by a booster dose of the COVID-19 vaccine at the beginning of the third trimester of pregnancy, once the magnitude of the maternofetal transfer is increased in this period.

There are many studies showing the presence of neutralizing anti-SARS-CoV-2 IgA, IgM, and IgG antibodies in breast milk of vaccinated women and women previously infected by COVID-19 (48, 51, 53, 65–67). An interesting study developed by Gray et al. addressed the magnitude of generated immunity post-vaccine (Pfizer or Moderna) in lactating women, which showed an increased level of virus-specific IgG after the vaccination and a high antibody level transferred to the neonate through breastfeeding, although the levels of IgA did not increase in breast milk, as expected, after the boost. In this context, these researchers concluded that IgG titers dominate in the breast milk of women who received the COVID-19 vaccine, whereas IgA titers dominate in the breast milk of women with previous SARS-CoV-2 infection (48). These results are in consonance with a prospective cohort study in Spain, which also found specific anti-SARS-CoV-2 IgG antibodies in breast milk after Pfizer vaccination (with levels even higher after the second dose) (65), but contrasts with a study by Valcarce et al., who demonstrated that after mRNA COVID-19 vaccination (Pfizer or Moderna), there was a predominance of SARS-CoV-2 IgA in human milk when compared to SARS-CoV-2 IgG levels (68).

To evaluate the duration of vaccine immunity, Perl et al. performed a cohort study including 84 lactating women and analyzed a total of 504 samples of breast milk collected before administration of the Pfizer vaccine and then, once a week—starting 2 weeks after the administration of the first dose—for 6 weeks. They found elevated levels of anti-SARS-CoV specific IgA during the follow-up: 61.8% of antibody positivity in the breast milk samples 2 weeks after the first dose; more than 85% positivity of these antibodies after week 4 (1 week after administration of the second dose of vaccine) and about 65.7% positivity at week 6 (67). Additionally, this same study analyzed anti-SARS-CoV IgG in the samples after vaccination and observed that the antibody levels remained low during the first 3 weeks, started to increase at week 4 (91.7% of samples testing positive for anti-SARS-CoV-2 IgG), and reached the peak at weeks 5 and 6 (97% of positivity). Therefore, considering the vigorous secretion of SARS-CoV-2-specific IgA and IgG in breast milk for at last 6 weeks after mRNA vaccination, these authors suggested that vaccination of lactating women offered protective effects against COVID-19 in the newborn (67). Similar findings were found by a recent Brazilian study that evaluated the presence of anti-SARS-CoV-2 IgA antibodies in human milk samples of women who received the CoronaVac vaccine, with the two doses of the vaccine administered 4 weeks apart. It was observed that the levels of anti-SARS-CoV-2 IgA started to increase in the first 2 weeks after the first dose of the vaccine, and they were significantly higher 5–6 weeks after vaccination (66).

Another study, developed by Perez et al., analyzed human milk samples from 27 women collected three times at 1, 3, and 6 months after they received the BNT162b2 (Pfizer) vaccine (25 of 27 women) or mRNA-1273 (Moderna) vaccine (2 of 27 women) (69). Concerning IgM antibodies, 7 of 24 (29.1%) women were positive after 1 month, 6 of 27 (22.2%) were positive after 3 months, and after 6 months post-vaccination, these antibodies were not detectable in breast milk. IgG antibodies were positive in breast milk samples from 24 of 24 (100%) lactating women in the first month, 25 of 27 (92.6%) in the third month, and 9 of 12 (75.0%) in the sixth month. On the other hand, 12 of 24 (50%) lactating mothers were positive for SARS-CoV-2-specific IgA 1 month after vaccination, 7 of 27 (25.9%) were positive at 3 months, and at 6 months’ IgA levels were not detected at significant levels above the baseline. The authors also evaluated the neutralizing activity of the cited antibodies and found that 20 of 24 (83.3%) breast milk samples showed neutralizing capacity at 1 month; 19 of 27 (70.4%) had neutralization activity at 3 months; only 3 of 12 (25.0%) maintained this neutralizing activity by month 6. In other words, they concluded that COVID-19 mRNA vaccination induced the production of SARS-CoV-2-specific antibodies for at least 6 months after vaccination, and neutralizing antibodies persisted for at least 3 months (69).

There is a lack of research that evaluates the efficiency of anti-SARS-CoV-2-specific IgM, IgG, and IgA transfer from breast milk to the infant’s serum (62, 75). A study by Yeo et al. analyzed the serum of 5 infants (age 3 to 20 months) of vaccinated women that were breastfeeding; a single serum sample was collected at a median of 48 days after their mothers received the second dose of BNT162b2 vaccine, and the researchers observed that there were no neutralizing antibodies detected in their serum (76). These results were also observed by Golan et al. in a study that did not identify anti-SARS-CoV-2 IgG antibodies in the plasma of infants whose mothers were vaccinated with mRNA-based vaccines for COVID-19 (mRNA-1273 and BNT162b2) during lactation (53). Additionally, a longitudinal cohort study by Schwartz et al. detected SARS-CoV-2 IgG in the oral mucosa of 3 of 5 (60%) breastfed infants of lactating women who were vaccinated against COVID-19 with the BNT162b2 messenger RNA vaccine but also did not find these antibodies in the infants’ serum (77). Therefore, further studies are needed to better understand these points.

## Cellular Immune Response Post-COVID-19 Vaccine in Pregnant Women

Besides humoral response, it is known that cellular immune response mediated by T cells is crucial for the combat of SARS-CoV-2 infection: while CD4^+^ T cells are important to develop specific antibodies against the virus, CD8^+^ T cells have a role in the identification and destruction of infected cells (62). A study that evaluated the participation of cellular immunity in the lactation of women vaccinated against COVID-19 revealed that after vaccination with COVID-19 mRNA, non-pregnant, pregnant, and lactating women had an increase in anti-SARS-CoV-2 CD4^+^ and CD8^+^ T cell counts, and this immune response was more robust to vaccine than to natural infection (52). In this context, another study with pregnant women who received the Pfizer vaccine showed that although the concentration of their antibodies against the virus decreased after several months of vaccination, their memory CD4^+^ and CD8^+^ cells continued to express proinflammatory cytokines (such as IFN-*γ*, TNF-α, and IL-2), which indicates that vaccination in pregnant women, as in other individuals, provides long-term protection against SARS-CoV-2 (78).

### Maternal Immune Cells in Human Milk and Cellular Immunity Transmission

The development of the newborn immune system starts *in utero* and is highly boosted by passive immunization through breastfeeding (79). Faced with an immature adaptive immune system that has not had the time to build up the necessary repertoires of cell clones and memory to permit the neonatal defense, the newborn takes into account immune cells and other defense components coming from breast milk. Of these components, we can highlight the high amounts of antibodies (mainly IgA), cytokines and other proteins (primarily lactoferrin) transferred from mother to child, and components of maternal cellular immunity such as macrophages, polymorphonuclear neutrophils, and lymphocytes (composed by approximately 83% of T cells and 4%–6% of B cells) (80–83). Breast milk lymphocytes are very abundant at delivery, decline over the first month postpartum to a steady state, and persist for up to 2 years (84–87). This was confirmed by a flow cytometry study that identified and quantified the CD45^+^ leukocyte populations in human breast milk and found cells like myeloid precursors, neutrophils, immature granulocytes, CD16^+^ and CD16^−^ monocytes, non-cytotoxic T cells, cytotoxic T and NK cells, eosinophils, basophils, B-cell precursors, and B cells (87). It is already known that the leukocytes are able to survive in the environment of the child’s digestive tract; reach the blood, lymph nodes, spleen, and other tissues/organs; and phagocytize and fight against pathogens (81, 82). Therefore, the properties offered by this group of cells provide active immunity to the infants, besides stimulating the achievement of their own immunocompetence (83).

Additionally, evidence shows that there are a few differences between breast milk and blood leukocytes: breast milk T cells and macrophages have more motility than those in blood, and colostrum lymphocytes have effector functions that can be transferred through breast milk and benefit the infant to respond against threats (82). Therefore, the properties offered by this group of cells provide active immunity to the infants, besides stimulating the achievement of their own immunocompetence (83).

The presence of memory T cells in human milk suggests that these cells were transferred from the mother to the infant to provide a rapid response against specific pathogens until their immune system becomes fully operative. In fact, studies demonstrated transferred breast milk memory CD4^+^ and CD8^+^ T cells in infants’ Peyer’s patches, spleen, and bone marrow; it is known that T lymphocytes in the intestine of neonates are recent thymic emigrants (progenitors of mature naive T lymphocytes) (80, 83); therefore, these specific memory T cells probably originated from their mothers through lactation. Sabbaj et al. analyzed a group of virus-infected lactating women and demonstrated that cytomegalovirus (CMV), influenza virus, Epstein–Barr virus (EBV), and HIV-specific CD8^+^ T cells were found in the breast milk. This suggests that an effector memory phenotype of CD8^+^ T cells is passed through breastfeeding to the newborns (88). Although studies about memory T cells against SARS-CoV-2 in human milk are scarce, Armistead et al. observed that the lactating breast contains a distinct T-cell population that can be modulated by maternal vaccination with potential implications for infant passive protection. These researchers have identified SARS-CoV-2 spike-specific T cells in mRNA vaccinated in lactating women (89). Another study conducted by Gonçalves et al. involving lactating women who received mRNA vaccination found a combination of spike-reactive T cells and anti-SARS-CoV-2 secreted IgA in their milk, which shows that immune transfer to the infant could linger even after weaning, especially because of long-lived memory T cells transferred (64). Such evidence points to the great importance of maternal vaccination, especially for the SARS-CoV-2 virus, as a cellular immunization strategy for the newborn through lactation.

## Other Bioactive Compounds in Human Milk

Human milk has a list of maternal immunomodulatory, antiviral, and anti-inflammatory elements that helps in the development of the newborn’s immune response (79). Recent research has shown that the risk of severe viral respiratory infections in infants is negatively associated with the duration of breastfeeding (90). Therefore, a crucial role in human milk is played by other components in addition to IgA, such as oligosaccharides, proteins (such as lactoferrin), lipids, and pro- and anti-inflammatory factors (TNF-α, interleukin-1 [IL-1], interleukin-10 [IL-10], prostaglandins E2 [PGE2], etc.) (91–93).

There is a lack of studies on the antivirals’ effects of breast milk against SARS-CoV-2, but some authors suggest that newborns can be protected from COVID-19 by milk proteins like lactoferrin, casein, and immunoglobulins, which have antiviral effects (94). It has already been reported that lactoferrin enhances NK cell activity, promotes neutrophil aggregation and adhesion, and blocks the SARS-CoV from entering host cells during the infection (93); it is likely that these findings could also be applied to SARS-CoV-2. Indeed, these breast milk bioactive molecules can have their immune response amplified after women’s vaccination, as has already been evidenced in the immunization against human rotavirus (95).

## Conclusion

The peculiarities of the immune system during pregnancy are one of the reasons pregnant women are included in a higher risk group for respiratory infections. With the emergence of the COVID-19 pandemic and the uncertainties around it, the concerns around pregnant women increased, mainly due to the possibility of maternal–fetal transmission of the virus. In this review, we assessed that the safety and efficacy of the developed COVID-19 vaccines did not differ between pregnant, lactating, and non-pregnant women. Furthermore, besides reducing the risks of post-COVID-19 complications, the benefits of vaccinating these groups are not restricted to them, since the production of neutralizing antibodies against SARS-CoV-2 by the mother can be transmitted to the fetus. Several studies showed that immunized women can transmit anti-SARS-CoV-2 IgG through the placenta, as has been confirmed by the presence of these antibodies in cord blood or the newborn serum after birth. Additionally, the seroprotection during the beginning of the infant’s life can be boosted by early third-trimester vaccination of their mothers, seeing that the magnitude of the maternal–fetal transfer is increased in this period. The passive immunization of the newborn also happens through breastfeeding; studies demonstrated the presence of anti-SARS-CoV-2 specific IgA, IgM, IgG, and T cells in the breast milk some weeks after a mother’s vaccination. These findings add even more benefits to breastfeeding, which naturally confers protection to infants due to the immunomodulatory, antiviral, and anti-inflammatory molecules that compose the human milk. Therefore, more studies involving pregnant and lactating women are needed to better characterize the vaccine immunogenicity among these populations. These results may help to create public health policies and to optimize the vaccine schedule, considering the durability of post-vaccine immunity, to ensure maternal–fetal protection against COVID-19.

## Author Contributions

ALA, APMM, and DMC performed an extensive review of literature about issues contemplated in the manuscript. ALA wrote the manuscript. JMVC, JELV, LDB-P and DMC reviewed the intellectual content and also helped to draft the manuscipt. JMVZ, JELV, and LDB-P conceived the proposal of the mini-review. All authors approved the final manuscript.

## Funding

This work was partially supported by the Post graduate Program in Biosciences and Physiopathology (PBF-UEM), by the Laboratory of Immunogenetics at Maringa State University (Proc. nº. 1589/2017-CSD-UEM), and by Brazilian funding agencies for scientific research, such as CAPES and CNPq.

## Conflict of Interest

The authors declare that the research was conducted in the absence of any commercial or financial relationships that could be construed as a potential conflict of interest.

## Publisher’s Note

All claims expressed in this article are solely those of the authors and do not necessarily represent those of their affiliated organizations, or those of the publisher, the editors and the reviewers. Any product that may be evaluated in this article, or claim that may be made by its manufacturer, is not guaranteed or endorsed by the publisher.
